# The BJGP Open Top 10 Most Read Research Articles of 2022: an editorial

**DOI:** 10.3399/BJGPO.2023.0026

**Published:** 2023-03-02

**Authors:** Alexander Burrell, Hajira Dambha-Miller

**Affiliations:** 1 BJGP Open, Royal College of General Practitioners, London, UK

**Keywords:** general practice, general practitioners, primary healthcare

It has been a busy and successful year for BJGP Open. As editors we have received a vast array of wonderful and interesting manuscripts. These have spanned diverse subject matters and locations from gestational diabetes in Norway to workforce retention in South Africa. In this editorial, we reflect on the articles that have resonated most with our readership, as highlighted by our recent list of *Top 10 Most Read Research Articles of 2022*.

The past few years have seen unprecedented change in how we consult in primary care. With remote consulting having moved from the default policy, at least in most European countries, in 2020 to media pariah in 2021,^1^ this year saw more balance with practices weighing up the potential risks and benefits of telehealth, and restructuring services to suit a ‘post-pandemic’ world. Several of our articles reflect the importance of human interaction, a foundation of our specialty, which was heavily impacted both professionally and personally by the pandemic. Verma and Kerrison rapidly reviewed patients’ and physicians’ experiences with remote consultations,^2^ finding that — although these can be more convenient than in-person appointments — patients and practitioners in this study acknowledged the impact of the loss of physical and visual assessment and non-verbal communication. The impersonal nature of remote consulting also meant that rapport and relationship building was challenging. Similarly, in Wanat *et al*’s^3^ qualitative exploration of perspectives on consultations for respiratory tract infections in eight European countries during the first wave of COVID-19, remote consultations were acceptable but the doctor–patient relationship was diminished. This is important when considering the ‘hidden pandemic’ of long COVID highlighted by Brennan *et al* ^4^ in their scoping review of management. Alongside traditional approaches to symptom identification and treatment, and facilitation of access to specialist services where needed, human interaction was once again highlighted, with GPs demonstrating concern, empathy, and understanding being key to optimal care. How effectively this can be done remotely is unclear.

What we do know is that remote consulting affects GPs’ behaviour: in Mayne *et al*’s^5^ exploration of sedentary behaviour among GPs, 80.7% of those surveyed reported an increase in sitting time compared to pre-pandemic, with 94.5% of those citing remote consulting as a reason for this. With this study showing an average of over 10 hours of sedentary time on a normal working day, the health of our workforce should be considered in future remote consulting system models. Active workstations may be a solution, though these were only available to 5.6% of those surveyed.

How we use language matters whether we are consulting remotely or in person. Parry *et al* ^6^ qualitatively investigated how people with osteoarthritis perceive flares: they found the term was often used to describe severe, infrequent, unpredictable episodes of pain but that this was applied inconsistently and not universally, with the term having *‘no clear fixed meaning’*.

The literature on whether remote consulting alters workload is still unclear. However, as GP consultation numbers rise and workforce shortages continue, clinically efficient, safe, and cost-effective care solutions are imperative. The importance of cost saving has been shown in Sampson *et al*’s^7^ article on the effects of a population-wide rollout of *Sleepio,* a program delivering digital cognitive behavioural therapy for insomnia. Through a reduction in primary care contacts and prescriptions for insomnia, a saving of £6.64 per patient was achieved. When scaled up, a potential saving of £20 million was suggested in the first year. Saran *et al*’s^8^ review found that tablet splitting could be safe, with the exception of sustained release tablets. The authors suggest that there is limited evidence to support previous concerns and propose that it may be an effective tool for using minimum effective doses and reducing medication costs. This may be of interest to patient-facing pharmacists in primary care, who Mann *et al* ^9^ found were trusted by patients to deliver expert medication advice and reviews, and had a positive impact on medicines use, including deprescribing and improvement in adherence.

As the primary care workforce changes, with allied health professional roles becoming more prevalent, two values are key: collaboration and sustainability. Two of our top 10 articles are examples of international collaboration from a European perspective, including van der Velden *et al*’s^10^ prospective audit of prescribing for respiratory tract infections in 18 European countries, which found that antibiotic prescribing varied by country, and was related to illness severity, comorbidity, age, and fever. GPs were confident of their prescribing decision in nearly 90% of consultations. Point of care testing was not associated with antibiotic prescribing, but the authors report it may enhance antibiotic stewardship in safely reversing confident prescriptions made on clinical grounds alone. For general practice to be sustainable and to continue to produce meaningful research to improve patient care, recruitment and retention are imperative. Stephenson *et al* ^11^ surveyed GP trainees and trainers to identify barriers and facilitators to primary care research involvement. Some of those surveyed wanted to take up academia, with a further 44% planning a career involving education. Barriers to involvement in research included a lack of role models in trainer roles, with funding for time and qualifications a key facilitator. Somewhat reassuringly when compared to recent British Medical Association surveys,^12^ only 2% of those surveyed were considering leaving medicine. As we look forward to the rest of 2023, keeping the next generation of GPs inspired and excited about primary care is a worthwhile goal to work towards for all involved.

BJGP Open is proud to be a journal for international primary care, early career researchers, and those working on the front line. 2022 was a productive year: our readership and social media following continued to grow and we received submissions from 32 countries across five continents. Our *Top 10 Most Read Research Articles of 2022* showed that as we move forward from the COVID-19 pandemic, remote consulting remains high on the academic and clinical primary care agenda. Questions remain around this, with many practices having to make their own decisions in the absence of clear central guidance about what is the ‘right’ balance of remote and in-person care to provide from both clinical and patient perspectives, what conditions and presentations can be managed safely remotely, and what triage tools should be used. We look forward to receiving submissions addressing these and other important questions in the coming year.
